# Radiographic outcomes decline linearly with increased time to surgery in distal radius fractures: A cohort analysis

**DOI:** 10.1177/17531934251379171

**Published:** 2025-09-29

**Authors:** Mats Wadsten, Albert Christersson, Ana Farah-Mwais, Magnus Tägil, Emma Haskovec, Markus Engquist, Viktor Schmidt

**Affiliations:** 1 Department of Diagnostics and Intervention (Orthopaedics), Umeå University, Sweden; 2 Department of Surgical Sciences/Orthopaedics, Uppsala University, Sweden; 3 Department of Clinical Sciences Lund, Orthopedics, Lund University, Sweden; 4 Department of Orthopedics, Ryhov Hospital, Sweden; 5 Department of Clinical Sciences at Danderyd Hospital, Karolinska Institutet, Sweden

**Keywords:** Delayed, distal radius fracture, outcomes, radius, surgery, timing

## Abstract

**Introduction:**

The optimal timing for distal radial fracture fixation remains controversial. Most previous studies have dichotomized timing into early or delayed categories, potentially obscuring the true effect of delay. This study investigated surgical timing as a continuous variable to determine its influence on radiographic alignment.

**Methods:**

In a retrospective multicentre cohort study, we reviewed 691 surgically treated distal radial fractures across four Swedish hospitals. Radiographic parameters assessed included dorsal tilt (primary outcome), radial inclination, ulnar variance, intra-articular step, coronal shift and anterior apposition. Logistic regression was used to analyse overall acceptable alignment, while linear regression was used for dorsal tilt. Models were adjusted for age and sex. Interobserver reliability was evaluated with intraclass correlation coefficients.

**Results:**

The mean patient age was 61 years, and 80% of the cohort were female. Each additional day delay to surgery increased the risk of unacceptable alignment by 6%, corresponding to a twofold risk increase with a 2 week delay. Dorsal tilt worsened linearly by approximately 0.34° per day, accumulating to nearly 5° after 2 weeks. Male sex was associated with significantly greater dorsal tilt (mean difference >2°) and reduced correction compared with females. Interclass correlation coefficients demonstrated excellent reliability for dorsal tilt (0.952) and radial inclination (0.947), and moderate reliability for ulnar variance (0.748) and coronal shift (0.611).

**Conclusion:**

A linear relationship was identified between surgical delay and declining radiographic outcomes, highlighting that each day’s delay progressively compromises fracture alignment. These findings emphasize the importance of prompt surgical intervention for distal radial fractures to achieve optimal radiographic results.

**Level of evidence:**

**III**

## Introduction

The incidence of distal radial fractures (DRFs) is rising, and the proportion treated surgically is increasing (Jerrhag et al., 2017; Viberg et al., 2023). Despite their prevalence, considerable debate remains regarding the timing for surgical intervention. Some studies suggest that early intervention improves functional outcomes (Khan et al., 2023; Sirniö et al., 2019). Others report that it may reduce complications (Keçeci et al., 2024; Yoon et al., 2020). However, several reports find no significant difference between early and delayed fixation (Grier et al., 2024; Howard et al., 2021; Lee et al., 2022).

Most research has focussed on functional outcomes, with relatively few studies examining the effects of surgical timing on radiographic results (Campbell et al., 2023). Furthermore, previous studies have often categorized surgical timing using arbitrary cut-off points rather than analysing it as a continuous variable, potentially oversimplifying its effect on patient outcomes (Grier et al., 2024).

To address this gap, this study aimed to investigate the effect of time to surgery as a continuous variable on radiographic outcomes in a large cohort of patients with distal radial fractures.

## Patients and method

### Study design and setting

The retrospective cohort study was conducted across four hospitals in Sweden, representing four of the six main healthcare regions. Data were collected by senior orthopaedic surgeons or supervised registrars. The ethics committee waived the requirement for informed consent owing to the retrospective nature of the study (Dnr 2021-00593, 2021-03212).

### Study population

The study population included all patients aged 18 years or older with a DRF visible on initial radiographs during the two separate years 2019 and 2023. Patients were identified using the ICD codes for DRF (S52.5 and S52.6) from digital medical records. The expected sample size was approximately 2000 patients, with each hospital contributing around 500 cases. To ensure an even distribution of cases over time, larger hospitals included only the first 21 patients per month.

Exclusion criteria included the absence of an initial radiograph before reduction or within 9 days post-injury, residence outside the hospital’s catchment area, previous fracture with malunion, closed reduction and casting before the initial radiograph and isolated AO type A1 fractures.

### Radiographic assessment

Radiographic images were evaluated according to national guidelines (Schmidt et al., 2022) and included measurements of dorsal tilt (primary outcome parameter), radial inclination, ulnar variance and intra-articular involvement. Coronal shift was measured on the anteroposterior projection. Anterior apposition (LaMartina et al., 2015) was assessed as displacement of no more than one cortical width in the lateral projection. Acceptable alignment for patients was defined according to functional demand (Mellstrand Navarro, 2021; Schmidt et al., 2022) (Table 1).

**Table 1.** Acceptable alignment.

	High functional demand	Intermediate functional demand
Dorsal tilt (deg)	<10	<20
Anterior tilt (deg)	<15	<15
Radial inclination (deg)	>15	>10
Ulnar shortening	<2 mm	<3 mm
Intra-articular step	<2 mm	<2 mm
Volar cortex	Aligned*	Aligned*
Coronal shift	<2 mm	—
DRUJ	Congruent	Congruent

* Defined as displacement in the anteroposterior direction of no more than one cortical width.

Radiographs were assessed postoperatively. Measurements were taken from the anteroposterior and lateral radiographs in neutral rotation. Measurement reliability was assessed by comparing measurements from consultant orthopaedic surgeons and residents in orthopaedic surgery, using the intraclass correlation coefficient (ICC).

### Data collection

Patient data were extracted from medical records, including age, gender, functional demand level, treatment type and time from injury to surgery.

### Statistics

The primary analysis examined the relationship between surgical timing and radiographic outcome, particularly dorsal tilt measured on postoperative radiographs. Logistic regression was used to assess overall acceptable alignment as the outcome, including dorsal tilt, radial inclination, ulnar variance, intra-articular step, coronal shift and anterior apposition. Linear regression modelling was used for dorsal tilt as the outcome variable. Sex and age were included in both models as covariates. Normality was assessed for with histograms and quintile–quintile plots. Continuous variables were evaluated for nonlinearity using ANOVA. No nonlinearity was confirmed and we therefore performed linear analyses. Interrater reliability for radiographic measurements was assessed using the ICC with ICC3: a two-way mixed effects model.

All analyses were performed with R 4.3.2, using the rms-package (v. 6.7-1) for regression modelling and contrasts, knitr (v. 1.45) for reproducible research, ggplot2 (v. 3.5.1) for plots and Gmisc (v. 3.0.3) with Greg (v. 2.0.2) for table output.

## Results

Of 1946 patients with DRFs, 693 were managed surgically. Complete data were available for 691 patients who were included in the analysis. The mean age was 61years and 80% were female. Most patients had acceptable alignment after surgical treatment (80%) and the mean time to surgery was about 1 week. Average radiological alignment was generally not far from anatomical alignment (Table 2).

**Table 2.** Base characteristics of surgically treated fractures.

	Female (*n* = 552)	Male (*n* = 139)	Overall (*n* = 691)
Age			
Median (IQR, range)	66 (18, 18–93)	50 (28, 18–85)	63 (20, 18–93)
**Acceptable alignment**			
Yes	438 (79.3%)	114 (82.0%)	552 (79.9%)
No	114 (20.7%)	25 (18.0%)	139 (20.1%)
**Time to surgery (days)**			
Median (IQR, range)	7 (8, 0–39)	6 (8, 0–27)	7 (8, 0–39)
Postoperative anterior tilt* (deg)			
Median (IQR, range)	5 (9, −20–29)	3 (9, −17–17)	5 (9, −20–29)
**Postoperative ulnar variance† (mm)**			
Median (IQR, range)	−0.9 (2.4, −7.0–4.6)	−1.4 (2.3, −6.0–4.1)	−1.0 (2.4, −7.0–4.6)
**Postoperative radial inclination (deg)**			
Median (IQR, Range)	20 (5, 5–34)	20 (5, 10–28)	20 (5, 5–34)
**Postoperative coronal shift (mm)**			
Median (IQR, range)	0 (0, 0–4.2)	0 (0, 0–3.2)	0 (0, 0–4.2)
**Surgical method (*n*, %)**			
Anterior plate	492 (87.9)	98 (69.8)	590 (84.2)
External fixator	6 (1.1)	5 (3.6)	11 (1.6)
Pins	29 (5.3)	5 (3.6)	34 (4.9)
External fixator and pins	13 (2.4)	7 (5.0)	20 (2.9)
Dorsal plate	1 (0.2)	6 (4.3)	7 (1.0)
Other	11 (2.0)	18 (12.9)	29 (4.2)

* Negative numbers would represent a dorsal tilt. Around 11 is anatomic alignment.

† Negative numbers would represent a shorter ulna compared to radius. Around −1 is anatomic alignment.

**Figure 1.**
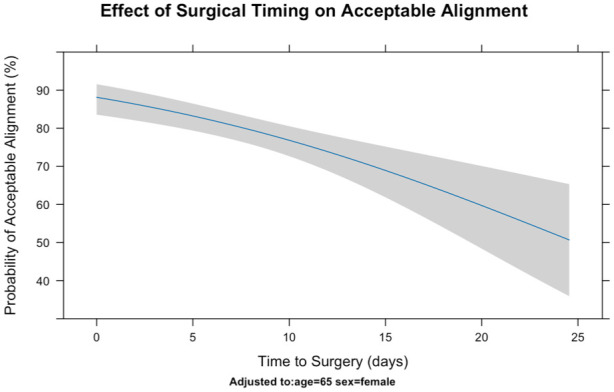
Effects of surgical timing on postoperative acceptable alignment (Table 1) in a logistic regression.

Overall acceptable alignment was analysed using logistic regression. Increased time to surgery was significantly associated with poorer alignment, whereas age and sex were not. Each additional day to surgery increased the risk of unacceptable alignment by 6%, representing a doubling of risk with a 2 week delay (Table 3; Figure 1; Supplementary Table 1).

**Table 3.** Odds ratio of acceptable alignment* by variable, in a logistic regression model.

Variable	Crude		Adjusted	
	OR	2.5–97.5%	OR	2.5–97.5 %
Time to surgery (days)	**1.08**	**1.04–1.12**	**1.08**	**1.04–1.12**
Age	1.01	0.99–1.02	1.01	0.99–1.02
Sex (male)	0.84	0.52–1.36	0.96	0.57–1.61

Bold denotes significance at *p* ⩽ 0.05.

* See Table 1.

**Figure 2.**
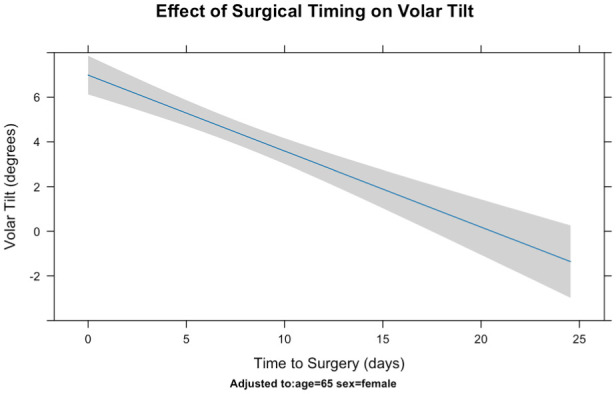
Effects of surgical timing on final postoperative anterior tilt in a linear regression.

Dorsal tilt was analysed separately using linear regression. Increased time to surgery and male sex were significantly associated with increased dorsal tilt, whereas age was not. For each additional day to surgery, dorsal tilt increased by 0.34°, representing almost 5° of increased dorsal tilt with a 2 week delay (Table 4; Figure 2). Male patients had a mean of over 2° more dorsal tilt than females.

**Table 4.** Variation in dorsal tilt by variable, in a linear regression model.

Variable	Crude		Adjusted	
	Coefficient	2.5–97.5%	Coefficient	2.5–97.5%
Time to surgery (days)	**−0.33**	**−0.42 to −0.24**	**−0.34**	**−0.43 to −0.25**
Age	0.01	**−**0.02 to 0.04	**−**0.01	**−**0.04 to 0.03
Sex (male)	**−1.85**	**−3.06 to −0.65**	**−2.05**	**−3.31 to −0.79**

Bold font denotes significance at *p* ⩽ 0.05.

These findings suggest that delay to surgery and male sex are associated with greater postoperative dorsal tilt, whereas patient age does not appear to influence this outcome.

**Figure 3.**
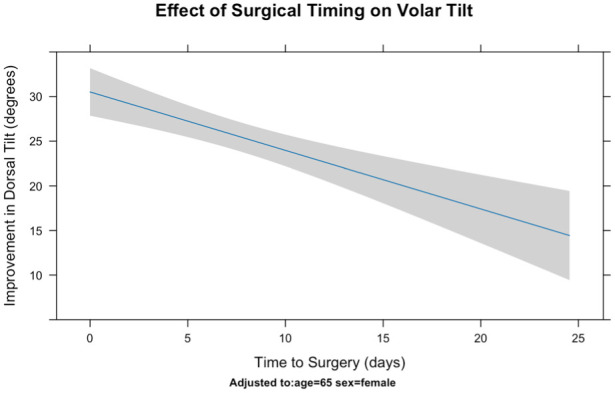
Effects of surgical timing on total improvement in anterior tilt in a linear regression.

A sensitivity analysis was performed using linear regression to examine predictors of improvement in dorsal tilt after surgery compared with the initial displacement. Increased time to surgery and male sex were associated with a reduced degree of improvement (Table 4; Figure 3). For each additional day the total restoration of dorsal tilt decreased by 0.66°, amounting to a reduction of almost 10° after a 2 week delay. Male patients were also found to have 7° less restoration of dorsal tilt than female patients.

The ICC was excellent (0.952) for dorsal tilt, excellent (0.947) for radial inclination, moderate (0.748) for ulnar variance and moderate for coronal shift (0.611).

## Discussion

The main finding of this study is that a longer time from fracture to surgery is associated with worse radiographic outcomes in DRFs. A 2 week delay doubles the risk of unacceptable alignment with an average increase of 5° dorsal tilt. Since the relationship is linear, a threshold cannot be defined. Patients should therefore be treated as early as possible. Interestingly, fractures operated on early had greater initial displacement yet still achieved better final alignment. These results support recommendations for early intervention to optimize fracture reduction and potentially improve outcomes.

Fracture healing begins with hematoma formation. Within days, a soft callus of fibrous tissue and cartilage starts developing (Einhorn and Gerstenfeld, 2015; Marsell and Einhorn, 2011). By around 14 days, early callus and soft tissue contractures make closed reduction nearly impossible, and open reduction may require removal of the callus (Wijffels et al., 2012). Delayed surgery may also impair vascularity and prolong healing (Freeland and Luber, 2005).

Other studies have examined the relationship between surgical delay and outcome. Hooper et al. (2021) found better surgeon-perceived reduction quality and less procedural difficulty in surgery performed within a week, although radiographic measurements were not included. However, others have shown that surgery after 2 weeks can still give acceptable outcomes (Lee et al., 2022; Wijffels et al., 2012). While previous studies have categorized surgery as early or delayed using fixed cut-off time points, we analysed surgical timing as a continuous variable, reducing the risk of Type I error (Altman and Royston, 2006) and to better assess the impact on radiographic outcomes. Our findings demonstrate a linear correlation between surgical timing, dorsal tilt and overall alignment, suggesting that prioritizing patients with unstable fractures may improve outcomes.

Campbell et al. (2023) found that late surgery was often performed by more experienced surgeons, suggesting potential selection bias, with fracture complexity and surgeon availability contributing to delays. We found, however, that fractures operated on early had greater initial displacement and better final alignment, reinforcing the benefits of early surgery (Figure 3). The reason for the worse radiographic outcomes in men remains unclear, but may reflect higher-energy injuries with more complex fracture patterns, making realignment more challenging.

Delayed surgery has also been associated with complications and worse patient-reported outcomes (Ashdown et al., 2021; Khan et al., 2023). Two randomized, controlled trials reported better outcomes with early surgery, although the delayed surgery groups were small (Mulders et al., 2019; Sirniö et al., 2019). Other studies report no long-term differences (Howard et al., 2021; Julian et al., 2023; Yamashita et al., 2015).

Radiographic alignment, particularly dorsal tilt, is the strongest predictor of function after a DRF (Schmidt et al., 2023; Wilcke et al., 2007). Howard et al. (2021) found no differences in the patient reported wrist evaluation between early and delayed surgery, but noted that delay to surgery may hinder reduction and affect outcomes. Our results support this finding, and support timely surgery to avoid malalignment and potential functional limitations.

The strengths of this study include the large patient cohort and the continuous analysis of surgical timing. This provides a more precise picture of how delay affects alignment compared with previous studies using broad time categories. The multicentre design across four Swedish hospitals represents clinical practice across different healthcare regions.

This study has limitations. Functional outcomes, such as grip strength or patient satisfaction, were not assessed. In addition, limiting inclusion to the first 21 patients per month in the larger hospitals may have introduced selection bias by increasing the relative proportion of patients from smaller hospitals, potentially affecting the ability to generalize the results. Moreover, we did not analyse complications or the experience level of the operating surgeon, which may have influenced the reduction quality for late surgeries (Landgren et al., 2017; Campbell et al., 2023). Future research should focus on long-term follow-up, including grip strength, range of motion and patient-reported outcome measures to confirm whether a delay in time to surgery also leads to functional deficits.

In conclusion, delay to surgery for a DRF increases the risk of overall unacceptable fracture alignment. Since previous research has linked dorsal tilt to functional impairment, our findings emphasize the importance of early surgical intervention. These results support recommendations for timely surgery and suggest that prioritizing patients with unstable fractures may help improve outcomes.

## Supplemental Material

sj-docx-1-jhs-10.1177_17531934251379171 – Supplemental material for Radiographic outcomes decline linearly with increased time to surgery in distal radius fractures: A cohort analysis Supplemental material, sj-docx-1-jhs-10.1177_17531934251379171 for Radiographic outcomes decline linearly with increased time to surgery in distal radius fractures: A cohort analysis by Mats Wadsten, Albert Christersson, Ana Farah-Mwais, Magnus Tägil, Emma Haskovec, Markus Engquist and Viktor Schmidt in Journal of Hand Surgery (European Volume)
