# Supplementary material for: Radiographic outcomes decline linearly with increased time to surgery in distal radius fractures: A cohort analysis
Source: J Hand Surg Eur Vol. 2025 Sep 29;51(2):173–8. doi: 10.1177/17531934251379171 (PMC12868306; doi:10.1177/17531934251379171)
Supplement: sj-docx-1-jhs-10.1177_17531934251379171 – Supplemental material for Radiographic outcomes decline linearly with increased time to surgery in distal radius fractures: A cohort analysis [file sj-docx-1-jhs-10.1177_17531934251379171.docx]

| **Supplementary table 1.** Total number of patients with unacceptable alignment* and the radiographic parameters in which they were unacceptable. | |
| --- | --- |
| **Parameter** | **n (%)** |
| Any unacceptable alignment | 134 (100) |
| Dorsal tilt | 28 (21) |
| Ulnar variance | 18 (13) |
| Radial inclination | 74 (55) |
| Continuity of volar cortex | 36 (27) |
| Intra-articular step-off | 5 (4) |
| Coronal shift | 3 (2) |
| Bold denotes statistically significant values.  *See Table 1. | |
